# A Retrospective Assessment of Referrals Between the Mental Health Liaison Team and Memory Assessment Service; Does Delayed Referral Due to Delirium Lead to Some Patients Being Lost to Follow-Up?

**DOI:** 10.1192/bjo.2024.368

**Published:** 2024-08-01

**Authors:** Thomas Fyall, Bethany Thompson, Ross Overshott

**Affiliations:** Pennine Care NHS Foundation Trust, Oldham, United Kingdom

## Abstract

**Aims:**

The assessment, diagnosis, and management of memory problems in older adults are routinely undertaken by memory assessment services (MAS) typically following referral from a GP. Mental health liaison teams (MHLT) newly identify many older people in acute hospitals with memory problems. Delirium is often diagnosed acutely and should be managed prior to any consideration of dementia diagnoses, however many of these people still have histories which also suggest underlying undiagnosed dementia. Referral policies advise of 3 months delay between delirium and MAS review to avoid misdiagnosis of dementia. MHLT therefore often request GP to refer at 3 months if still indicated. It is felt that some patients may be lost to follow-up via this route; our aim was to explore this further with a view to establishing a more robust direct referral pathway if indicated.

**Methods:**

Electronic records of patients under the care of MHLT aged over 65 from June 2022 to June 2023 were reviewed. This excluded patients who were referred and discharged from MHLT after a single assessment. We collected retrospective data for 8 months during this 12-month period. For any patients with memory concerns, we recorded where MAS referral was recommended and whether they were subsequently referred and seen.

**Results:**

108 patients over the age of 65 under the care of MHLT were identified. 69 patients had memory problems, 28 of whom already had established diagnoses or were already under MAS and 41 had newly identified memory problems. Of these 41 patients, 15 were felt to need MAS referral due to possible dementia. 3 were referred directly to MAS by MHLT and were seen. 5 were later referred to MAS by GP on MHLT recommendation and were seen. 7 were not later referred to MAS despite it being recommended.

**Conclusion:**

All 3 patients whom MHLT were able to refer directly to MAS were seen, whereas 7 out of 12 (58%) patients for whom 3-month delayed referral by GP was requested were not seen. The policy of 3-month delay avoids misdiagnosis due to delirium, but in practice also leaves some patients with missed opportunities for diagnosis and management of dementia. There is a need for a more robust delayed referral pathway to memory assessment services from mental health liaison teams. We hope to use these findings to improve our local referral pathways and share this information to support other localities.

